# Transcriptomic analysis of elderly women with low muscle mass: association with immune system pathway

**DOI:** 10.18632/aging.203505

**Published:** 2021-09-07

**Authors:** Levi H. Jales Neto, Bidossessi W. Hounkpe, Georgea H. Fernandes, Liliam Takayama, Valéria F. Caparbo, Neuza H.M. Lopes, Alexandre C. Pereira, Rosa M.R. Pereira

**Affiliations:** 1 Bone Metabolism Laboratory, Rheumatology Division Faculdade de Medicina FMUSP, Universidade de Sao Paulo, Sao Paulo, SP, Brazil; 2 Instituto do Coracao (InCor), Hospital das Clinicas HCFMUSP, Faculdade de Medicina, Universidade de Sao Paulo, Sao Paulo, SP, Brazil; 3 Laboratory of Genetics and Molecular Cardiology, Instituto do Coração (InCor), Hospital das Clinicas HCFMUSP, Faculdade de Medicina, Universidade de Sao Paulo, Sao Paulo, SP, Brazil

**Keywords:** low muscle mass, muscle weakness, immune system, vitamin D, aging

## Abstract

Despite the well-established association of gene expression deregulation with low muscle mass (LMM), the associated biological mechanisms remain unclear. Transcriptomic studies are capable to identify key mediators in complex diseases. We aimed to identify relevant mediators and biological mechanisms associated with age-related LMM. LMM-associated genes were detected by logistic regression using microarray data of 20 elderly women with LMM and 20 age and race-matched controls extracted from our SPAH Study (GSE152073). We performed weighted gene co-expression analysis (WGCNA) that correlated the identified gene modules with laboratorial characteristics. Gene enrichment analysis was performed and an LMM predictive model was constructed using Support Vector Machine (SVM). Overall, 821 discriminating transcripts clusters were identified (|beta coefficient| >1; *p*-value <0.01). From this list, 45 predictors of LMM were detected by SVM and validated with 0.7 of accuracy. Our results revealed that the well-described association of inflammation, immunity and metabolic alterations is also relevant at transcriptomic level. WGCNA highlighted a correlation of genes modules involved in immunity pathways with vitamin D level (R = 0.63, *p* = 0.004) and the Agatston score (R = 0.51, *p* = 0.02). Our study generated a predicted regulatory network and revealed significant metabolic pathways related to aging processes, showing key mediators that warrant further investigation.

## INTRODUCTION

Accelerated loss of muscle mass is one of the dramatic changes associated with aging and reflects muscle remodeling in elderly population [1, 2]. Age-related low muscle mass leads to lower mobility, disability and increased mortality in elderly population [1, 2]. The diagnosis is particularly based on the evaluation of appendicular lean mass (ALM) by dual X-ray absorptiometry (DXA). Among the methods used to identify LMM, the criterion defined by Newman et al. in 2003 [1] is based on ALM measurement adjusted for total fat mass [3, 4]. Therefore, it seems to identify more individual with sarcopenia, particularly those with sarcopenic obesity [3, 4]. In a previous study, we define the Newman residual specific to our population and demonstrated that LMM is a risk factor for increased mortality in elderly men and women [4]. In our Brazilian community a residual that yielded the best performance of sensitivity and specificity to define LMM in elderly women was −1.32 [4].

Multiple factors are involved in the development of age-related low muscle mass, including several known age-related factors, such as neuromuscular degeneration, abnormal protein turnover, decreased hormone levels and sensitivity, chronic inflammation, oxidative stress, and lifestyle (nutritional status and physical inactivity) [5]. Despite the well-described contribution of these factors, current therapies are only limited to lifestyle changes by mainly targeting resistance training and/or protein supplementation [6, 7]. There is no drug specifically approved for sarcopenia treatment [8]. Owing to the high prevalence of the clinical complications of LMM in elderly women, further studies are needed to identify possible effective therapies. Thus, defining key cellular and molecular mechanism underlying this condition is a crucial step to identify new relevant therapeutic targets. However, these mechanisms are not fully elucidated. We believe a global transcriptomic portrait of the systemic implications of LMM would advance our understanding of the biological processes associated with this condition, thereby paving the way to more efficient clinical intervention.

So far, the pathophysiological mechanisms of muscle mass loss in elderly women are not fully understood. Several studies have already pointed the contribution of genetic associations to low muscle mass process and its complications [9]. Application of transcriptomic technologies to muscle of sarcopenia patients has already identified key alterations of biological processes specific to human and murine muscle tissue [10, 11]. However, most studies have focused on local perturbations, rather than systemic implications of LMM at transcriptomic level.

In this study, we aim to identify gene sets and biological processes that differentiate elderly women with LMM from age-matched controls. Our study identified predictors of LMM at transcriptomic level and revealed the molecular mechanisms underlying the disease. Gene co-expression analysis revealed gene modules correlated with relevant laboratorial and clinical data of the patients, providing rather than a classical transcriptomic profile analysis, relevant clinical information associated with LMM gene expression profiles.

## RESULTS

### Sample characteristics

Using the definition of Newman et al. [1] and the residual previously defined for our population, we included forty elderly women in the study, of which 20 had LMM (residual <−1.32) and 20 age and race-matched controls without LMM. Lower weight, total and appendicular lean mass and lower BMI were observed in the LMM group (Table 1). No difference in abdominal circumference was found between the groups. A higher percentage of fat was observed in the group with LMM (median 41.63 (3.94) vs. 38.61 (4.18); *p* = 0.02 for LMM and controls respectively). Regarding associated chronic diseases and medications for chronic use, no differences were found between groups. Comparing the biochemistry between the groups, a significantly lower serum level of vitamin D (25OHD) (18.75 (7.06) vs. 24.15 (6.74) ng/ml; *p* = 0.018) and serum insulin (11.6 (5.7) vs. 17 (14.57) UI/mL; *p* = 0.02) was observed in the LMM group. The participants included in this study are homogeneous in terms of lifestyle and habits. They reside in the same geographic region, and have the same socioeconomic, educational level and access to the same health system. As part of the SPAH cohort (São Paulo Ageing and Health Study) they were followed up in the city of São Paulo, and are routinely guided by medical doctors, medical students, and other health professionals from the University of São Paulo (USP), School of Medicine. Serum albumin levels were similar in both groups (4.65 ± 0.2 vs. 4.65 ± 0.22 g/dL; *p* = 0.30 for LMM and control respectively) and were in the normal range (3.5–5.2 g/dL) suggesting a reasonable nutritional level. Regarding consumption of milk and derivative products, no difference was observed between the groups with LMM and control (median 527.1 vs. 553.2 mg/day; *p* = 0.75 for LMM and control respectively). All other characteristics are presented in Table 1.

**Table 1:** Clinical, anthropometric, biochemical and calcium score characteristics of the participants.

**Variables**	**LMM *n* = 20**	**Control *n* = 20**	***p*-value**
Clinical variables			
Age, years, mean (SD)	80.45 (4.44)	79.65 (3.76)	0.54
Ancestry, *n* (%)			
Caucasian	10 (50)	10 (50)	1
Black	10 (50)	10 (50)	
BMI, kg/m^2^, mean (SD)	28.17 (4.26)	32.68 (5.08)	**0.004**
Physical activity, *n* (%)			
Low	5 (25)	0 (0)	**0.047**
Moderate	10 (50)	16 (80)	
High	5 (25)	4 (20)	
Hypertension, *n* (%)	16 (80)	15 (75)	1.0
Diabetes Mellitus, *n* (%)	6 (30)	3 (15)	0.45
Dyslipidemia, *n* (%)	9 (45)	12 (60)	0.53
Heart attack, *n* (%)	1 (5)	2 (10)	1.0
Stroke, *n* (%)	2 (10)	1 (5)	**1.0**
Osteoporosis, *n* (%)	8 (0.4)	11 (0.55)	0.53
Hypothyroidism, *n* (%)	4 (20)	4 (20)	1.0
Alcohol intake, *n* (%)	2 (10)	3 (15)	1.0
Smoking, *n* (%)	1 (5)	1 (5)	1.0
Medication			
AAS, *n* (%)	7 (35)	7 (35)	1.0
ACE inhibitors, *n* (%)	5 (25)	8 (40)	0.50
Beta blocker, *n* (%)	6 (30)	8 (40)	0.74
Thiazide diuretic, *n* (%)	10 (50)	6 (30)	0.33
Calcium antagonists, *n* (%)	2 (10)	6 (30)	0.23
Statins, *n* (%)	7 (35)	11 (55)	0.34
Antidepressants, *n* (%)	4 (20)	0 (0)	0.10
Bisphosphonates, *n* (%)	8 (40)	8 (40)	1.00
Corticoid, *n* (%)	1 (5)	0 (0%)	1.00
Vitamin D supplement, *n* (%)	9 (45)	12 (60)	0.53
Lean and Fat Mass			
Total lean mass, g, mean (SD)	34294.25 (4738.27)	42698.73 (5344.11)	**<0.0001**
Appendicular lean mass, Kg, mean (SD)	13.13 (2.25)	18.26 (2.53)	**<0.0001**
Fat mass, g (SD)	26192.1 (5900.05)	27641.25 (9434.72)	0.31
Fat, % (SD)	41.63 (3.94)	38.61 (4.18)	**0.02**
Newman Residual, median (SD)	−2.77 (1.47)	1.55 (1.88)	**<0.0001**
Laboratory variables			
25OHD, ng/mL, mean (SD)	18.75 (7.06)	24.15 (6.74)	**0.018**
Total calcium, mg/dL, mean (SD)	9.86 (0.54)	9.61 (0.36)	0.09
iPTH, pg/dL, median (IQR)	61 (29.75)	50.5 (17)	0.48
Alkaline phosphatase, U/L, median (IQR)	78 (31.25)	70 (37.25)	0.43
Total phosphorus, mg/dL, mean (SD)	3.48 (0.32)	3.51 (0.37)	0.78
Creatinine clearance, mL/min/1.73m^2^, median (IQR)	54.45 (16.65)	58 (37.82)	0.50
Total cholesterol, mg/dL, mean (SD)	210.95 (39.86)	206.40 (41.34)	0.72
LDL cholesterol, mg/dL, mean (SD)	123.35 (39.62)	127.30 (41.74)	0.76
HDL cholesterol, mg/dL, mean (SD)	59.75 (14.88)	52.60 (16.53)	0.17
Triglycerides, mg/dL, mean (SD)	139.20 (42.13)	150.75 (64.66)	0.51
Lipoprotein A, mg/dL, median (IQR)	67 (97)	50 (67.5)	0.93
Blood glucose, mg/dL, median (IQR)	101 (29.25)	93 (15.5)	0.62
Serum insulin, UI/mL, median (IQR)	11.6 (5.7)	17 (14.57)	**0.02**
C Reative Protein, mg/L, median (IQR)	1.9 (3.55)	2.4 (3.05)	0.52
Albumin, g/dL, mean (SD)	4.65 (0.2)	4.65 (0.22)	0.30
Ferritin, ng/mL, median (IQR)	131.65 (161.44)	192.6 (136.7)	0.2
Serum uric acid, mg/dL, mean (SD)	4.89 (1.15)	5.61 (1.71)	0.13
Agatston method			
0, *n* (%)	8 (0.4)	4 (0.2)	0.5
1–100, *n* (%)	4 (0.2)	6 (0.3)	
>100, *n* (%)	6 (0.3)	9 (0.45)	
ND	2 (0.1)	1 (0.05)	
Dietary intake			
Milk and derivatives^*^, mg/day, median (IQR)	527.1 (433.35)	553.2 (518.25)	0.75

The Shapiro-Wilk test was used to evaluate the normality. The values of p are for comparisons of means (Student's *t*-test), median (Mann-Whitney test) or proportions (Chi-square test or Fisher's test). Values of *p* < 0.05 were considered significant. Abbreviations: 25OHD: 25-hydroxyvitamin D; BMI: body mass index; iPTH: intact parathyroid hormone, IQR: interquartile range; SD: standard deviation; ND: non-determined. ^*^Milk and derivatives are presented as an estimated daily calcium ingested quantity.

### Discriminating transcript clusters and differential expression in LMM

**Figure 1:**
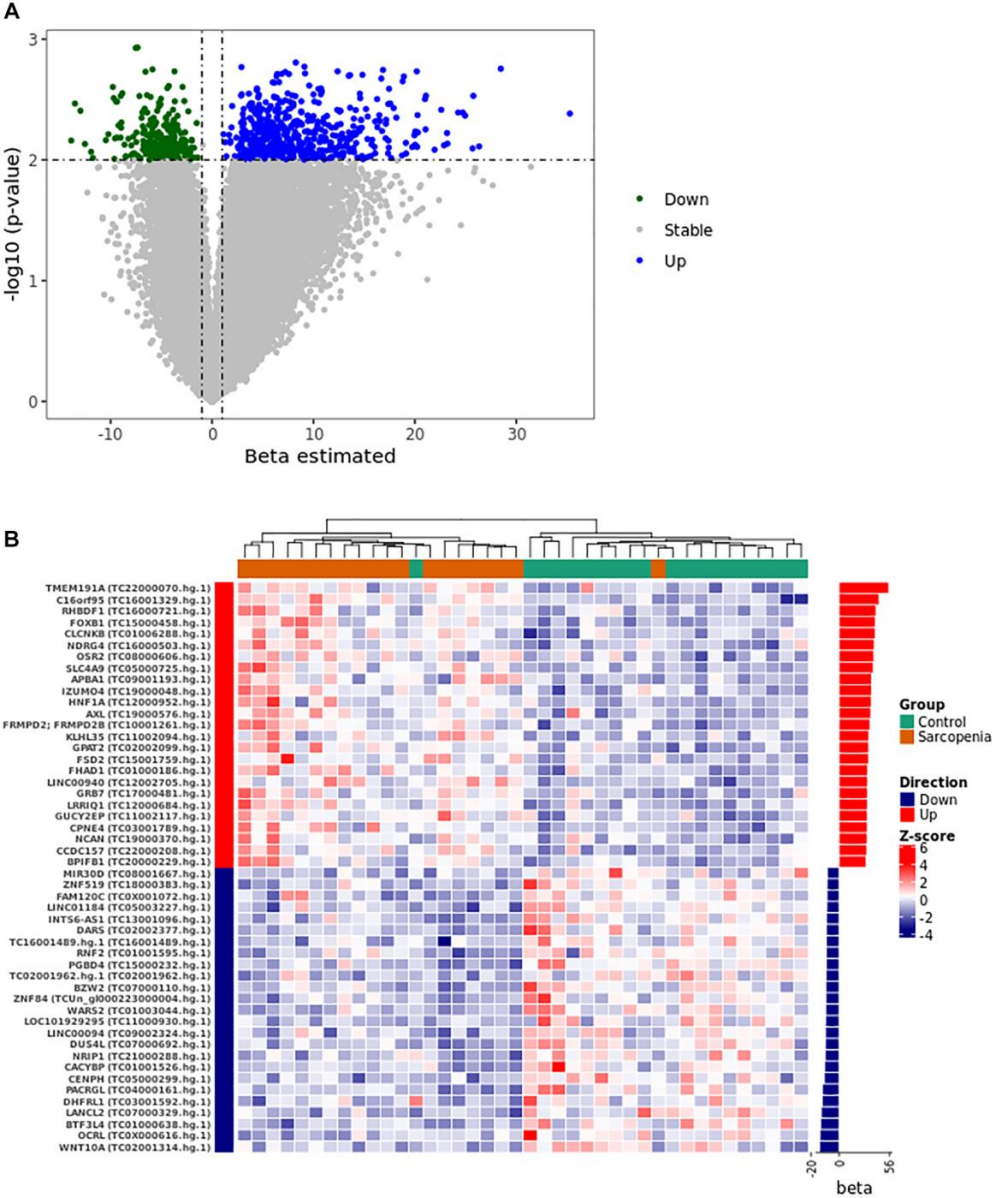
**Gene expression pattern from the logistical regression analysis.** (**A**) The volcano plot was constructed using the full list of 67528 transcript clusters analyzed. The top 821 transcripts were highlighted in blue (556 PPR) and green (255 PNR). A *p*-value of <0.01 and |beta| >1 was considered statistically significant. (**B**) The heat map shows the unsupervised clustering of the normalized expression pattern. The dendrogram indicates two clusters that stratified LMM and control group in distinct clusters. The right panel of the heat map shows the bar graph of beta coefficient values. Abbreviations: Beta: Estimated logistic regression coefficient; PPR: Predictor with Positive Relationship also called Up in panel **A** and **B**; PNR: Predictor with Negative Relationship also called Down in panel **A** and **B**.

In this analysis, for simplicity purposes, transcript clusters are sometimes identified by their corresponding genes symbols. The logistic regression model adjustment identified 821 unique transcript clusters of which 565 with positive coefficients (PPR) and 255 with negative coefficients (PNR) (Supplementary Table 1 and Figure 1A). Differential expression analysis identified 56 and 79 up- and down-regulated transcript clusters respectively (Supplementary Table 2). Among these transcript clusters, fifteen are also identified in the logistic regression model (Table 2). We then used unsupervised clustering of the normalized expression of the top 40 predictors to show their expression pattern between samples. This analysis stratifies the control and LMM groups in two different clusters (Figure 1B). The heat map of the expression profile shows a high homogeneity in each group when we consider this subset of 40 discriminating transcript clusters (Figure 1B).

**Table 2:** List of 15 differentially expressed transcript clusters identified in logistic regression model.

**Transcript Cluster**	**Ensembl Genes**	**Gene Symbol**	**Biotype**	**Beta**	**FC**
TC0X001828.hg.1	ENSG00000157600	TMEM164	Protein coding	1.29	1.82
TC0X000538.hg.1	ENSG00000157600	TMEM164	Protein coding	1.69	1.64
TC0X000540.hg.1	ENSG00000265584	MIR3978	miRNA	1.81	1.63
TC03001967.hg.1	ENSG00000200052	Y RNA	misc RNA	1.79	1.57
TC04001133.hg.1	ENSG00000252975	Y RNA	misc RNA	2.9	1.56
TC09000563.hg.1	ENSG00000200261	Y RNA	misc RNA	1.41	1.54
TC11000956.hg.1	ENSG00000282373	BIRC3	Protein coding	−2.21	0.64
TC08001180.hg.1	ENSG00000254165	AC090739.1	lncRNA	−2.17	0.62
TC01005703.hg.1	ENSG00000260948	AL390195.2	lncRNA	−2.91	0.61
TC0X002160.hg.1	ENSG00000166432	ZMAT1	Protein coding	−2.33	0.6
TC0X002160.hg.1	ENSG00000150347	ZMAT1	Processed Transcript	−2.33	0.6
TC10002092.hg.1	ENSG00000023445	ARID5B	Protein coding	−1.75	0.59
TC15000394.hg.1	ENSG00000244879; ENSG00000284284	GABPB1-AS1; MIR4712	LncRNA; miRNA	−1.52	0.56
TC14002234.hg.1	ENSG00000211923	IGHD3-10	IG D gene	−0.9	0.3

Transcript clusters were ranked according to the fold change. Abbreviations: FC: Fold-change; Beta: Estimated logistic regression coefficient.

### Support vector machine-based LMM classification model

RFE algorithm discriminates a list of the 45 most informative variables from the discriminating transcripts clusters identified in the logistic regression adjustment. This subset of transcript clusters was used to train a SVM model with a polynomial kernel of degree 1. Model performance was evaluated in the test cohort and shows an accuracy of 0.70. This list of 45 classifiers is enriched in differentially expressed transcript clusters based on their fold changes (Supplementary Table 3).

### Functional gene set enrichment highlights an alteration of muscle development and metabolism pathways in LMM

**Figure 2:**
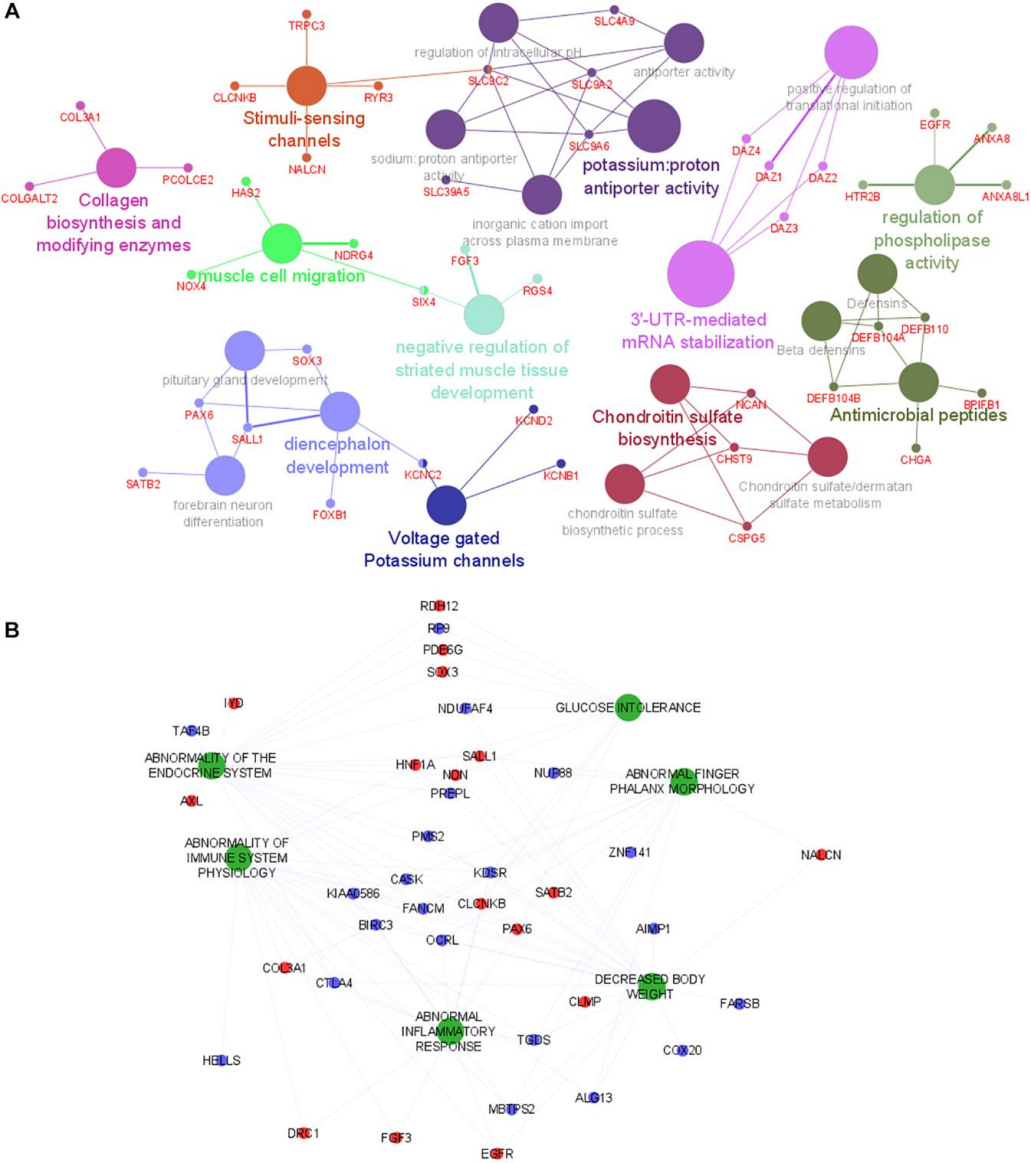
**Functional analysis of genes and human phenotypes associated with LMM.** (**A**) Pathways that are associated with the gene discriminated in the logistic regression (with positive coefficient) are indicated by colored nodes. GSA terms are interconnected with their associated genes. Related GSA terms are indicated by the same color. (**B**) The human phenotype ontologies are presented in green and shared genes are represented as red (positive coefficient) and blue (negative coefficient) dots. Abbreviation: GSA: Gene Set Analysis.

Genes associated with the subset of transcript clusters with positive and negative regression coefficients were then used to perform to different gene set analyses with ClueGO in Cytoscape software. Using the lists of annotated genes, ClueGO identifies and clusters biological pathways and gene ontology terms that participate in the same biological function, thereby removing redundancy. The top significant and non-redundant biological pathways and gene ontology terms associated with PPR genes were shown in Figure 2A. Muscle development pathways and metabolism ontology terms were overrepresented in this analysis. ClueGO shows few gene interactions between the different enriched gene set groups, suggesting an absence of hub genes involved in the simultaneous regulation of different pathways (Figure 2A). Genes with negative regression coefficients were associated with metabolism deregulation (Supplementary Figure 1). Even though these analyses provide us interesting information about the mechanisms underlying the LMM, they did not reveal the human phenotypes that were associated with the observed alterations.

### Human phenotype ontology (HPO) analysis reveals the involvement of immune and inflammatory alterations

To gain additional insights into the phenotypes previously associated with the LMM discriminating genes (identified in the logistic regression analysis), a HPO analysis was performed with the full list of predictors using GenePattern [12]. Figure 2B shows several relevant phenotypes up-regulated in LMM. Phenotypes associated with immune system deregulation, inflammation and pathologies involving muscle disfunction were overrepresented in the HPO network. Interestingly, these phenotypes are highly connected, suggesting a tight relationship between them and highlighting a probable association of their common mediators with the physiological mechanisms that underlie LMM development. In addition, based on network connectivity analysis, the following genes were predicted as hub genes of the HPO network: (i) CLCNKB and KDSR (abnormality of endocrine system, glucose intolerance, abnormality of immune system physiology, abnormal inflammatory response, decreased body weight); (ii) *KIAA0586* and *OCRL* (abnormality of endocrine system, abnormality of immune system physiology, abnormal inflammatory response, decreased body weight, abnormal phalanx morphology); (iii) *FANCM* (abnormality of endocrine system, abnormality of immune system physiology, decreased body weight, abnormal phalanx morphology); (iv) *TGDS* (abnormality of immune system physiology, abnormal inflammatory response, decreased body weight, abnormal phalanx morphology); (v) *NDN* (abnormality of endocrine system, glucose intolerance, abnormality of immune system physiology, decreased body weight). These genes are highly connected with most of the phenotypes and might play prominent role in the regulation of LMM.

### Prediction of the regulatory networks

**Figure 3:**
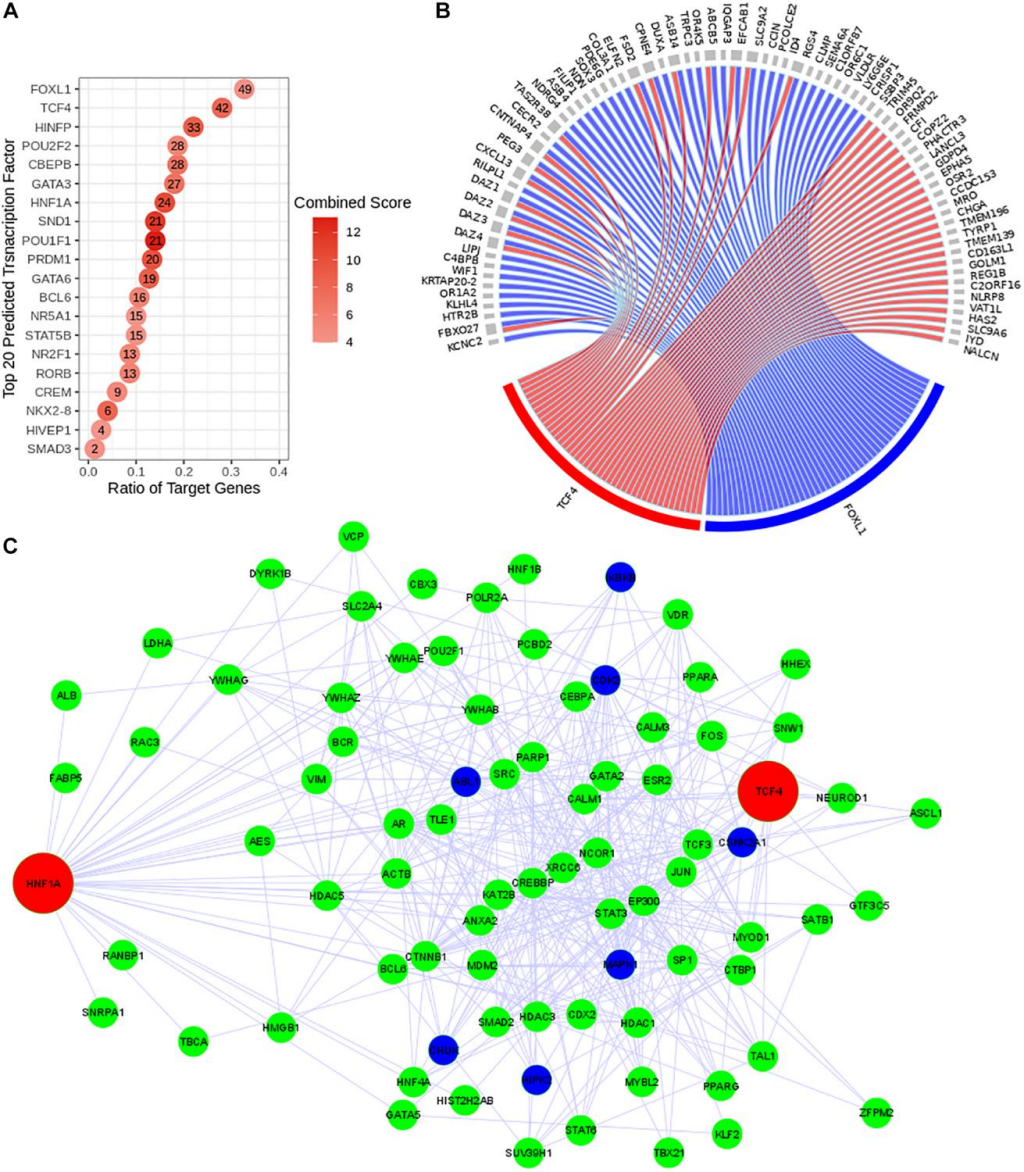
**Reconstruction of the predicted transcriptional regulatory network.** TF regulatory network was reconstructed using Expression2Kinase tool. (**A**) Top 20 predicted TFs were presented and ranked based on their combined scores. The intensity of the red coloration is proportional to the combined scores. The ratio of the target genes (x axis) indicates the proportion of genes targeted by a determined TF. (**B**) The circos diagram shows the interaction between the top two predicted TFs (*TCF4* in red and *FOXL1* in blue), ranked by the ratio of target genes) and their targeted genes (gray). Both TFs target 16 common genes. (**C**) Co-regulatory network of *TCF4* and *HNF1A*. The transcriptional regulatory network is presented with the transcription factors (TFs) in red, the intermediate protein that are predicted to interact with these TFs in green and the kinases in dark blue.

The regulatory network system upstream to the expression of the discriminating genes (with positive regression coefficients) was reconstructed using Expression2Kinase tool. This prediction tool allows us to identify in Figure 3A the top 20 predicted transcription factors (TFs) that can regulate the expression of the genes detected in this study (Figure 3A). The circos diagram in Figure 3B represents the relationship between the top 2 TFs that target the largest number of genes identified in our study (49 and 42 genes for *FOXL1* and *TCF4* respectively). We then analyzed the subnetworks of regulation and observed a common regulatory network pattern associated with *TCF4* and *HNF1A* that was identified as a PPR (estimated coefficient β = 22.87; *p*-value = 0.007). The regulatory network in Figure 3C presents these TFs (red nodes); (ii) the intermediate proteins likely to be involved in the formation of a regulatory complex with these TFs (green nodes) and (iii) the kinases that are expected to be the activators of the regulatory complexes (dark blue nodes). This predicted regulatory network connects *HNF1A* and *TCF4* and suggests a possibility of a co-regulatory system involving both TFs (Figure 3C).

### Gene modules detected by weighted gene co-expression network analysis are correlated with patient's characteristics

**Figure 4:**
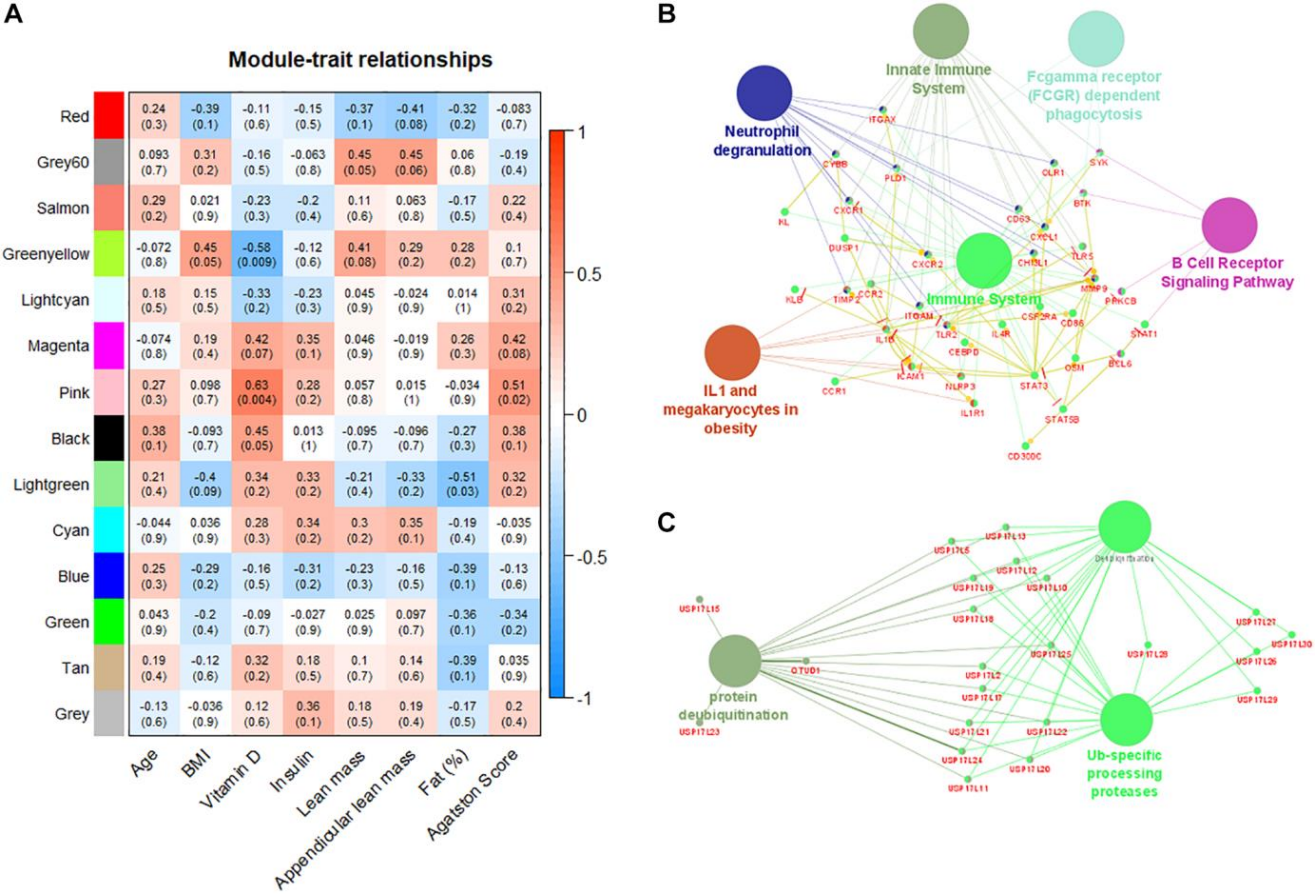
**Weighted gene co-expression network analysis reveals modules correlated with patient´s characteristics.** (**A**) Correlation matrix of detected gene modules with the characteristics of patients. Red color indicates positive correlation and blue color indicates negative correlation. Pink module is positively correlated with plasma levels of vitamin D and Agatston score. (**B**) Pink module is enriched with immune system pathways. (**C**) Green-yellow module is enriched with pathways associated with ubiquitination processes.

Fourteen co-expression modules were identified in the weighted co-expression analysis performed exclusively with the patient group. Correlation of clinical and laboratorial data with gene modules shows tight relationship with relevant parameters (Figure 4A). Pink module is moderately correlated with both serum levels of vitamin D (r = 0.63, *p*-value = 0.004) and Agatston score (r = 0.51, *p*-value = 0.02). This module regroups genes involved in immune system regulation including: (I) innate immunity, phagocytosis, and neutrophil degranulation, (ii) B cell receptor and IL1 signaling pathways (Figure 4B). In contrast, green-yellow and light-green modules are negatively correlated with plasma level of vitamin D and body fat percentage (r = −0.58, *p*-value = 0.009 and −0.51, *p*-value = 0.05 respectively). Both green-yellow and light-green modules are associated with ubiquitination mechanisms (Figure 4C). In addition, genes of green-yellow are involved in erythrocyte specific metabolism pathways and oxidoreduction processes (Supplementary Figure 1).

## DISCUSSION

Age-related LMM is a multiple factorial geriatric condition that is associated with known age-related conditions [5]. Despite its prevalence in older population and its impact on patients’ quality of life, cellular and biological processes associated with LMM are not fully investigated. This highlights the need for studies addressing in more detail the systemic molecular alterations in this condition to identify possible therapeutic targets. By performing transcriptomic analysis in elderly women with LMM, we were able to identify genes that discriminate LMM from matched controls and showed that alterations of muscle development, protein metabolism pathways, immune system and inflammation are involved in the pathogenesis of LMM. In addition, weighted gene co-expression network analysis revealed a correlation of gene module involved in immunity pathways.

Using human transcriptome microarray and a bioinformatic approach based on logistic regression analysis, we identified 821 transcript clusters (565 with positive coefficients (β coefficient >1 and *p*-value <0.01) and 255 with negative coefficients (β coefficient <−1 and *p*-value <0.01)) that discriminate LMM patients from age and race-matched controls. We took advantage of the performance of a machine learning method (Support Vector Machine) to further filter this list of predictors. Support Vector Machine identified 45 classifiers of LMM with an accuracy 0.7. This approach has already been used in other scenario to classifier complex diseases [13, 14] based on gene expression profile due to its effectiveness and best performance with high dimensional data such as transcriptomic data.

We observed that the identified genes by logistic regression are predominantly associated with muscle development and metabolic pathways (Figure 2A). In addition, human phenotypes associated with immune alteration and inflammation are also overrepresented (Figure 2B). These results reflect well-described perturbations associated with aging [15, 16] and have already associated with sarcopenia process and progress in several recent studies [6, 17]. Among the top ranked genes, there are two transcription factors (TF) *FOXB1* and *HNF1A* that can be involved in the regulation of key mechanisms underlying the process of LMM. Of note, using a regulatory network reconstruction approach, we identified a co-regulation network of *HNF1A* (Figure 3C) that is a TF with a wide range of functions [18] and *TCF4* (a predicted TF). Together these TFs target 16 common genes identified in our study. In pancreas ß-islet cells, *HNF1A* contributes to insulin secretion in response to glucose and is associated with maturity-onset diabetes of the young-like diabetes [19]. In our study we observed a low serum level of insulin in LMM group (11.6 vs. 17; *p* = 0.02). *TCF4* has been described as a key regulator of myogenesis [20]. However, their role in peripheral blood cells is uncertain. Thus, further detailed studies are necessary to investigate its contribution to LMM processes and progress.

Although cellular and molecular mechanisms associated with LMM are not understood, inflammatory alterations, immune senescence and protein metabolism have been suggested to be involved in this process [17, 21]. Ageing is generally associated with altered inflammation with increased levels of several pro-inflammatory markers such as CRP, TNF-α, IL-6 and IL-1 [22, 23]. Moreover, cross sectional studies have suggested the increase of IL-6 and TNF-a in sarcopenia [24]. In our study, the analysis of human phenotypes associated with LMM reveals great contribution of immune system and inflammation perturbations (Figure 2B). In addition, network analysis identified key hub genes (*CLCNKB, KDSR, KIAA0586, OCRL, FANCM, TGDS, NDN*) that make an inter-connection between most of the identified human phenotypes, suggesting their prominent role in the progression of LMM and a cross talk between immune-inflammation and muscle mass decline.

Moreover, we identified by weighted gene co-expression network analysis gene modules correlated with patients’ relevant clinical and laboratory characteristics. This approach is complementary to the classical differential expression analysis and have the advantage to associate gene expression profiles with patients data, improving the interpretability of the finding [25, 26]. The weighted gene co-expression analysis identified 14 correlated modules (Figure 4A). A gene module involving immune system alterations was positively correlated with serum levels of vitamin D (r = 0.63, *p*-value = 0.004) and Agatston score (r = 0.51, *p*-value = 0.02). Epidemiological studies have shown that a poor vitamin D status is associated with an increased risk of several diseases, including autoimmune diseases [27]. Of note, a lower serum level vitamin D (25-OH) was observed in the group of LMM women (18.75 vs. 24.15 ng/ml, *p* = 0.018), supporting the deterioration of the interplay between skeletal muscle and the immune system in this population [28]. An animal model suggests that vitamin D deficiency induces muscle waste and muscle protein degradation in ubiquitin proteasome pathway-dependent manner [29]. Interestingly, two gene modules negatively correlated with vitamin D serum levels and body fat percentage (r = −0.58, *p*-value = 0.009 and –0.51, *p*-value = 0.05 respectively) are also associated with ubiquitination mechanisms, confirming the critical participation of protein metabolism pathways in the progression of sarcopenia [30, 31].

This study has a main limitation worth noting. Our analysis included a relatively small sample, limiting the extrapolation of the obtained results to the general population of older women. However these participants were recruited from a highly admixed population [32] suggesting the presence of a diversified genetic background.

Our study provides new insights into the biological mechanisms and key mediators that can be involved in the development and/or progression of LMM in elderly women and highlights the contribution of vitamin D deficiency to age-related conditions. We identified lists of discriminating transcripts and weighted co-expression gene modules that can be involved in the pathogenesis of LMM by combining logistic regression analysis, a machine learning approach and network analysis. Finally, our results revealed that the well characterized association between inflammation, immunity and metabolic alterations and age-related muscle waste is also consistently observed at transcriptomic level. Noteworthy, our predicted models do not intend to highlight a relation of causality between the identified molecular interactions/mechanisms and the development of LMM. Instead, they provide a global view and new hypothesis-generating dataset, that could be explored in future investigations. To gain more insights into the mechanisms and regulatory interactions upstream to LMM development and progression, multiomics integrative approaches must also be considered in future studies.

## METHODS

### Subjects and microarray dataset

The subjects of this study were selected from the population-based survey (São Paulo Ageing and Health [SPAH] study) followed by the bone metabolism outpatient clinic of the Faculty of Medicine of the University of Sao Paulo (FMUSP). Transcriptome analysis of a total of 90 elderly women from this population were performed and described in our previous studies [33, 34] using microarray technology. From this microarray dataset publicly deposited in the GEO NCBI database under the accession number GSE152073, we selected 40 elderly women, of which 20 were with LMM (defined by a Newman's residual <−1.32) and 20 age and race-matched controls (residual >−1.32). Clinical and laboratory characteristics of this cohort were extracted from our database. Race was defined based on the self-reported race of second-generation ancestors, an approach previously used for the Brazilian population [35]. Physical activity was classified as (a) low, when not even housework is performed; (b) moderate, for regular housework, non-regular walking, gardening; and (c) high, for regular housework and daily regular physical activity at least twice a week for 30 min [36]. This research was approved by the local Ethics in Research Committee of the Medicine Faculty of São Paulo University/Brazil.

### Transcriptomic analysis

#### Microarray pre-processing and analysis

Microarray dataset was extracted from the repository São Paulo Aging and Health Study (SPAH), our previously published database [33, 34]. Raw microarray datasets were deposited in GEO NCBI repository at the accession number GSE152073. Normalization was performed by the Robust Multichip Average (RMA) method [37] using Transcriptome Analysis Console (TAC) software. Probes are summarized into a single probe set corresponding to a single transcript cluster. Then probe sets were annotated using biomaRt package [38] in R environment. Considering the binary variable Low of Muscle Mass (LMM), we adjusted a logistic regression model with age as a covariate in R to discriminate genes that affect this dependent variable [34]. Genes with regression coefficients β ≥ 1 or β ≤ −1 and p ≤ 0.01 were considered significant. In this approach a positive coefficient reflects an association with LMM. These discriminating variables were called predictor with positive relationship (PPR) in this study). Negative value reflects an association with LMM absence (predictor with negative relationship [PNR]). Additionally, a differential expression analysis was also performed using limma package to identify differentially expressed (DE) genes (transcript clusters) in the comparison of LMM vs. control group. We also applied the Empirical Bayes framework to estimate the more precise expression of each transcript cluster. All analyses were performed with R (version 3.7).

### Functional gene set analysis

To facilitate the interpretation of the biological relevance of discriminating genes selected from the logistic regression adjustment, a functional gene set analysis (GSA) was performed using ClueGO [39]. ClueGO, a plug-in of Cytoscape [40], predicts the functional gene ontology terms and biological pathways associated with our genes list. Then, it organizes the detected pathways in functionally grouped networks to highlight the relevant biological relationship between them. We applied a fusion criterion to reduce the redundancy of the terms that have similar associated gene sets. To further filter the most important terms and pathways, two-sided hyper-geometric distribution tests was used and terms at a significant level Bonferroni adjusted *p*-value of ≤0.05 were selected. To gain more insights into the human phenotype previously associated with the gene sets, a single-sample GSEA (ssGSEA) was also performed with GenePattern [12, 41] using the Human Phenotype Ontology (HPO) database [42]. Differentially activated HPO were then identified by comparing ssGSA score between LMM and control using RankProd package [43].

### Regulatory gene network prediction

To refine the exploratory analysis, the prediction of the regulatory networks of transcription factors (TFs) and kinases underlying gene expression of the PPR gene list was performed using Expression2Kinases (X2K) tool [44]. Only the top 20 list of human TFs and kinases ranked based on *p*-value were selected. Transcriptional regulatory network was visualized using Cytoscape software [40].

### Predictive model of LMM using support vector machine (SVM)

We submitted the discriminating transcript clusters identified by logistic regression to Recursive Feature Elimination (RFE) algorithm using e1071 and caret package. RFE was applied to the same subset of microarray dataset included in the logistic regression analysis (20 LMM and 20 controls) to select the most informative features (transcript clusters) for the SVM model training. The Training cohort was consisted of 70% of the SPAH dataset randomly selected and the remaining sample (test cohort) was used to validate the model. SVM model was trained with a polynomial kernel of degree 1. Model performance was evaluated based on its accuracy.

### Weighted gene co-expression network analysis (WGCNA)

Gene co-expression analysis was performed using WGCNA method [45] as previously described elsewhere [26]. We included in this analysis the 20 LMM patients previously analyzed in the logistic regression analysis. To construct the weighted gene co-expression networks, WGCNA determines the connection strengths between nodes (protein coding genes) by computing the pairwise correlations between their expression profiles and using a soft thresholding of the Pearson correlation. Modules of the weighted gene co-expression network constituted of groups of highly correlated genes were identified and correlated with relevant laboratorial and clinical data of the patients. Module highly correlated with patient's data were selected for further gene set enrichment analysis using ClueGO [39] plugin in Cytoscape [40].

## Supplementary Materials

Supplementary Figure 1

Supplementary Table 1

Supplementary Table 2

Supplementary Table 3
