# Supplementary material for: Transcriptomic analysis of elderly women with low muscle mass: association with immune system pathway
Source: Aging (Albany NY). 2021 Sep 7;13(17):20992–1008. doi: 10.18632/aging.203505 (PMC8457609; doi:10.18632/aging.203505)
Supplement: Supplementary Figure 1 [file aging-13-203505-s001.pdf]

SUPPLEMENTARY FIGURE

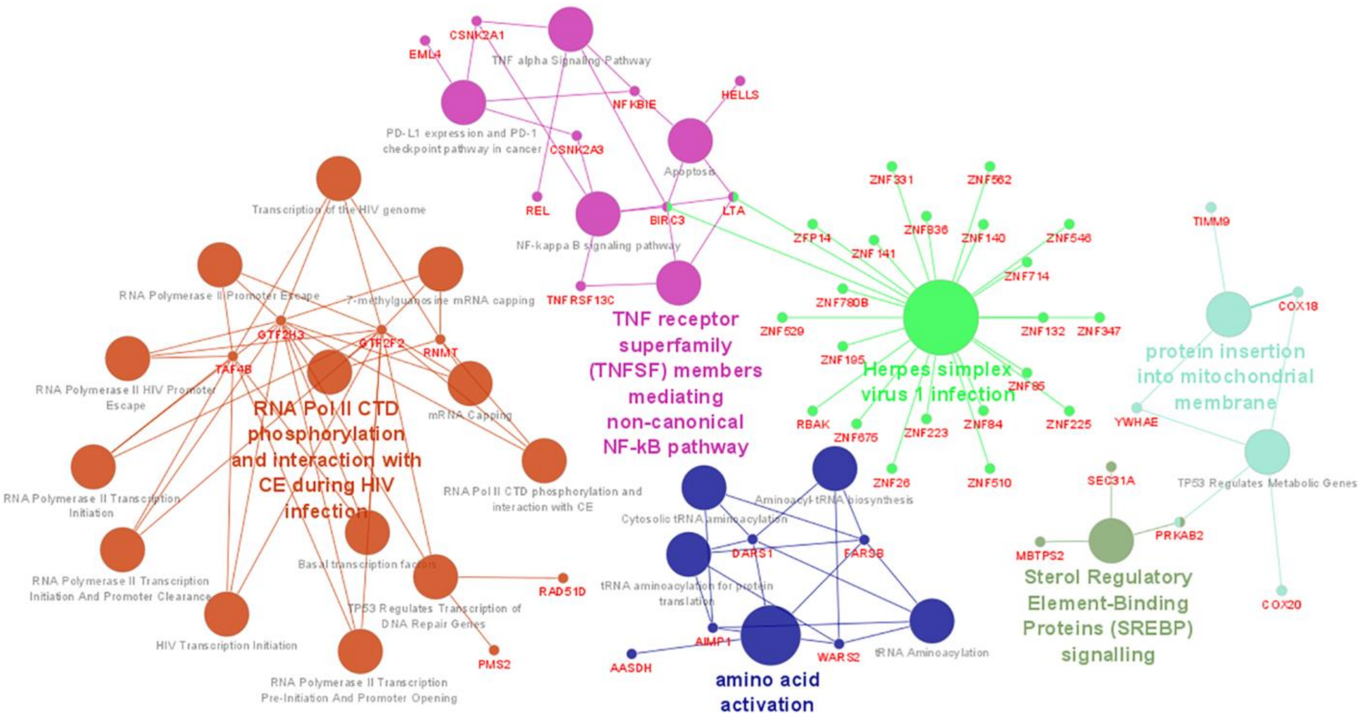

Supplementary Figure 1. Gene set analysis of genes with negative logistic regression. GSA terms are interconnected with their associated genes. Related GSA terms are indicated by the same color. Abbreviation: GSA: Gene Set Analysis.
